# RNA sequencing-derived gene co-expression and drug-gene interaction analysis reveal STAT1 as a potential therapeutic target in thrombotic antiphospholipid syndrome

**DOI:** 10.3389/fimmu.2026.1741872

**Published:** 2026-03-05

**Authors:** Michail Baltsiotis, Kleio-Maria Verrou, Petros P. Sfikakis, Maria G. Tektonidou

**Affiliations:** 1 First Department of Propaedeutic Internal Medicine, Joint Academic Rheumatology Program, Medical School, National and Kapodistrian University of Athens, Athens, Greece; 2 Center of New Biotechnologies and Precision Medicine, Medical School, National and Kapodistrian University of Athens, Athens, Greece

**Keywords:** antiphospholipid syndrome, gene co-expression network analysis, interferon, RNA sequencing, transducer and activator of transcription 1 (STAT1)

## Abstract

**Objective:**

Thrombotic primary antiphospholipid syndrome (PAPS) pathogenesis remains undefined, and recurrent thrombosis may occur despite adequate anticoagulation treatment. Identifying disease-specific molecular pathways and regulators can help in the discovery of novel therapeutic targets. Herein, we examine gene co-expression networks and potential druggable targets in thrombotic PAPS.

**Methods:**

We analyzed a whole-blood RNA-sequencing dataset from 62 well-characterized patients with thrombotic PAPS (40% with recurrent thrombosis), and 29 age/sex-matched healthy controls(HCs). Weighted Gene Co-expression Network Analysis (WGCNA) was performed to identify gene modules associated with PAPS, followed by enrichment analysis. Drug-gene interaction analysis of hub regulators within the identified networks was applied. Genes were classified based on target drug annotation and priority categories (low/medium/high).

**Results:**

WGCNA of whole-blood transcriptome of thrombotic PAPS and HCs, which included 8,190 expressed genes, identified five co-expression modules, two of which correlated with PAPS: the yellow, consisted of 42 genes enriched in immune-related functions, and the brown comprised 144 genes with a regulatory signature enriched in transcription activation pathways. A merged module demonstrated enhanced correlation with PAPS compared with HCs (r=0.221, p=0.035). Both yellow and brown, and merged module, were co-regulated by Transducer and Activator of Transcription 1 (*STAT1*), which emerged as a central hub gene. *STAT1* was also present in 5 of 6 immune-related pathways. In drug-gene interaction analysis, *STAT1* was among the four highly-ranked genes, and displayed many interactions and strong pharmacological support.

**Conclusion:**

*STAT1* is identified as a central regulator of gene expression networks in PAPS, integrating both immune-related and regulatory processes. Assessment of pharmacological target availability revealed *STAT1* as a promising treatment target.

## Introduction

Antiphospholipid syndrome (APS) is a systemic autoimmune disorder characterized by recurrent arterial and venous thrombosis, pregnancy morbidity, microvascular manifestations and the persistent presence of antiphospholipid antibodies (aPL), namely anticardiolipin antibodies, anti-β2 glycoprotein I antibodies, and lupus anticoagulant. APS is classified as primary (primary APS, PAPS) or in association with other systemic autoimmune disorders. Although APS primarily manifests in young adults, a 5% 5-year mortality rate has been described in large APS cohorts, with stroke and myocardial infarction being the primary causes of death (1, 2).

Previous knowledge of PAPS pathogenesis primarily focused on thrombotic and fibrinolytic mechanisms, and accordingly, therapeutic goals included anticoagulation and/or antiplatelet therapy as the mainstay treatment strategy. Advances in our understanding of PAPS pathophysiology reveal a complex interrelationship between inflammation and thrombosis mechanisms, termed thromboinflammation (3). Augmenting evidence has demonstrated that aPL can induce activation of endothelial cells and monocytes expressing tissue factor, oxidative stress, cytokine release, complement activation, platelet-neutrophil interaction with subsequent neutrophil extracellular trap (NET) formation, leading to thrombin generation and thrombus formation (4–9). However, the molecular regulators amplifying thromboinflammatory responses and their contribution to PAPS clinical presentation are not fully defined, and there is currently no specific targeted therapy for PAPS.

High-throughput transcriptomic profiling provides a powerful approach to identify the molecular mechanisms underlying systemic autoimmune diseases. RNA sequencing (RNA-seq), in particular, enables unbiased and comprehensive characterization of the transcriptome, allowing the identification of disease-specific signatures, cell-type-specific responses, and novel therapeutic targets (10). While microarray and sporadic RNA-seq studies have provided insights into PAPS biology across selected immune cell subsets (11), systematic analyses of the whole blood transcriptome networks that could lead to specific, druggable targets are lacking.

Weighted Gene Co-expression Network Analysis (WGCNA) is a bioinformatic method designed to explore correlation patterns among genes across transcriptomic datasets (12). Unlike traditional differential expression analysis, WGCNA identifies modules of co-regulated genes and relates them to clinical traits, offering insights into gene networks and key hub regulators that may drive disease phenotypes. This approach has been successfully applied in autoimmune disorders to uncover critical pathways and candidate biomarkers, but it has not yet been employed in PAPS (13, 14). Drug-gene interaction analysis using omics data and machine learning techniques, such as network analysis, has also been performed in some systemic autoimmune diseases, involving the identification of targets that are both “druggable” (can be modulated by a drug) and relevant to the disease pathophysiology (15).

In the present study, we applied WGCNA and drug-gene interaction analysis to whole-blood RNA-Seq data from patients with thrombotic PAPS and age- and sex-matched healthy controls (HCs), aiming to identify disease-associated gene co-expression networks, reveal central hub genes, and point out potential targets for therapeutic intervention.

## Patients and methods

### Patients

Our cohort consisted of 64 well-characterized adult patients with thrombotic PAPS followed at our department and 32 age- and sex-matched HCs recruited through institutional and hospital workplace advertisements (**Supplementary Table 1****).** All patients fulfilled the Sydney classification criteria for APS (16) as well as the 2023 ACR/EULAR criteria when they were applied to this cohort (17). Individuals meeting the classification criteria for systemic lupus erythematosus (SLE) (18) or other autoimmune disorders were excluded from the study. Preprocessing of the RNA-seq data has been previously described (19), and we utilized the resulting count matrix of 19,920 genes for the present analysis. During quality control for the WGCNA analysis, samples from 2 patients and 3 healthy controls were removed. In total, data from 62 thrombotic PAPS patients and 29 HCs were included in the WGCNA and drug-gene interaction analyses.

### Data processing and quality control

We applied gene-level filtering, retaining only genes with ≥15 raw counts in ≥75% of samples across the entire cohort (n=91). This filtering strategy builds on our previous work (19) with more stringent thresholds appropriate for WGCNA: the count threshold of 15 aligns with the recommended 10–15 counts for reliable expression estimates (20), while the 75% sample requirement ensures genes are consistently expressed across the majority of samples, which is essential for calculating reliable pairwise correlations in co-expression network analysis. This filtering resulted in 8,190 genes for downstream analysis.

### Normalization

Count data were normalized using the variance-stabilizing transformation (VST) implemented in DESeq2 (21). This transformation stabilizes variance across the range of expression values, making the data more suitable for correlation-based network analysis. In the batch-corrected expression matrix (19,920 genes x 91 samples), a small proportion of values (3,020; 0.17%) were negative, arising from the batch correction process; 91.5% of these were small values between -10 and 0. Gene filtering (>=15 counts in >=75% samples) removed most lowly-expressed genes where these negatives concentrated; remaining negative values were set to zero to meet DESeq2 input requirements. The filtered and normalized expression matrix (**Supplementary Table 2**) served as input for all subsequent analyses.

### Weighted gene co-expression network analysis

#### Network construction and module detection

Co-expression network analysis was performed using the WGCNA R package (version 1.72-1)12. To construct the weighted gene co-expression network, we first determined the appropriate soft-thresholding power using the pickSoftThreshold() function, testing powers ranging from 1 to 10 and 12 to 50 (by increments of 2). Based on the scale-free topology criterion (R² > 0.8) and connectivity preservation, we selected a soft-thresholding power of 14.

The co-expression network was constructed using the blockwiseModules() function with the following parameters: network type = “signed” (preserving positive and negative correlations), TOM type = “signed” (signed topological overlap matrix), maximum block size = 19,000 genes (sufficient for our dataset of 8,190 genes), minimum module size = 30 genes, deepSplit = 2 (moderate sensitivity for splitting modules), merge cut height = 0.25 (for merging similar modules), and random seed = 1234 (ensuring reproducibility). WGCNA parameters were selected to balance detection of distinct co-expression patterns with biological interpretability. These settings resulted in five distinct modules plus a large grey module (7,450 genes) containing genes without strong co-expression patterns, which is typical in whole blood transcriptome analyses of diseases characterized by modest transcriptional changes and clinical heterogeneity, such as PAPS. The analysis identified five distinct co-expression modules plus a grey module containing unassigned genes.

#### Module-trait association analysis

Module eigengenes (MEs), representing the first principal component of each module’s expression matrix, were calculated for all identified modules. We assessed the relationship between module eigengenes and clinical traits using Pearson correlation analysis. Statistical significance of correlations was determined using Student’s t-test, with p-values calculated for each module-trait association.

#### Module merging

Modules with significant positive correlation with APS, namely yellow and brown, were merged for further analysis (MEyb module). For merged modules, we recalculated module eigengenes and assessed module-trait correlations using the same statistical approach described above. The eigengene correlation between the yellow and brown modules (r = 0.20, dissimilarity = 0.80) exceeded the standard WGCNA merging threshold (dissimilarity < 0.25). These modules were therefore merged based on biological rationale rather than eigengene similarity: the yellow module containing genes enriched in immune response and IFN signaling and the brown module exhibiting enrichment in transcription regulation and RNA processing pathways, were the only two modules showing positive correlation with PAPS status, with the highest correlation coefficients among all colored modules (brown: r = 0.209, p = 0.046; yellow: r = 0.187, p = 0.076; both significant at α = 0.10). In addition to established evidence on the high type I and II IFN expression in PAPS (22), previous studies have also shown (23) that the splicing machinery was significantly altered in leukocytes from patients with PAPS, as well in those from SLE and SLE-APS, and it was distinctly linked to the pathophysiology of these closely related systemic autoimmune disorders. A strong relationship between the splicing machinery component and the IFN-related genes was also observed in SLE patients (23), indicating scientific evidence for merging these functionally connected modules. The merging decision was not made to optimize module-trait significance, but rather to combine potentially relevant pathways.

### Functional enrichment analysis

To elucidate the biological functions of identified modules, we performed comprehensive pathway enrichment analysis using the clusterProfiler R package (version 4.0) with the org.Hs.eg.db annotation database (24), for each module separately. Enrichment analyses were applied for Gene Ontology (GO) Biological Process, KEGG and REACTOME Pathways. Benjamini-Hochberg for multiple testing correction was applied, with adjusted p-values < 0.05 considered statistically significant.

### Network visualization

Co-expression networks were visualized using Cytoscape (version 3.9.1) (25) with the yFiles Organic Layout algorithm for optimal node positioning. For the MEyb network, we selected hub genes based on intramodular connectivity (kWithin) and displayed edges for gene pairs with correlation coefficients above the third quartile threshold.

Network nodes were annotated with pathway membership data for six significantly enriched pathways of interest identified through REACTOME enrichment analysis. Pathway-annotated genes were represented as pie chart nodes, with each slice corresponding to membership in a specific pathway.

#### Drug-gene interaction assessment

Assessment of drug-gene interactions and therapeutic targeting potential was performed using the Drug Gene Interaction Database (DGIdb) v5.0 (26), which aggregates drug-gene interactions from multiple curated databases, including DrugBank, ChEMBL, and other expert-curated sources. The analysis was conducted in R (v4.5.1) with the DGIdbGraphQL API to query drug-gene interactions and druggable genome classifications for 186 genes comprising the MEyb module. For each gene, we extracted the following drug-gene interaction metrics: (i) presence of known drug interactions, (ii) number of targeting drugs, (iii) number of documented interactions, (iv) interaction types (inhibitor, agonist, antagonist), (v) source databases, and (vi) druggable genome classification status. A composite drug-gene interaction score was calculated by integrating multiple factors: base score derived from the number of targeting drugs (weighted 40%), interaction type quality bonus for high-value interactions such as inhibitors and antagonists (30%), and source reliability bonus for expert-curated databases (30%). Priority scores were computed by combining drug-gene interaction scores (70%) with druggable genome status (30%) to rank genes for potential therapeutic targeting.

Genes were classified into three drug-gene interaction categories based on composite scores: Low (0-0.2), Moderate (0.2-0.5), and High (0.5-1.0). Similarly, priority classifications were assigned as Low Priority (0-0.3), Medium Priority (0.3-0.6), and High Priority (0.6-1.0). Network visualization was performed to explore relationships between the top-ranked genes, where connections were established based on similarity in drug-gene interaction profiles using correlation analysis of normalized feature matrices.

### Statistical analysis

All statistical analyses were performed in the R statistical computing environment (version 4.2.1). Pearson correlation coefficients were calculated for all gene-gene correlations and module-trait associations. Statistical significance was assessed using Student’s t-test with two-tailed p-values. The Benjamini-Hochberg false discovery rate (FDR) method was applied for multiple testing correction where appropriate, with FDR-adjusted p-values < 0.05 considered significant.

For module preservation and quality assessment, we calculated module membership (kME) values for all genes, representing the correlation between each gene’s expression profile and its module eigengene. Intramodular connectivity (kWithin) was computed to identify hub genes within each module.

## Results

The demographic, clinical, laboratory and treatment characteristics of 62 patients with thrombotic APS (all White people) are shown in **Supplementary Table 1**. Among the 29 healthy controls, 68.9% were females, and their mean (S.D.) age was 42.8 (10.6) (range: 26-62). Forty percent of APS patients had a history of recurrent thrombotic events, 78.3% had double aPL positivity and 51.6% had triple aPL positivity.

### Co-expression modules and correlations with thrombotic PAPS

Weighted Gene Co-expression Network Analysis (WGCNA) of the filtered whole blood transcriptomes, including 8,190 expressed genes, identified five co-expression modules. Genes were clustered using topological overlap dissimilarity, and modules were defined by dynamic tree cutting followed by merging of similar clusters, as shown in **Supplementary Figure 1A**. This analysis resulted in five distinct modules: the turquoise module (349 genes), the blue module (171 genes), the brown module (144 genes), the yellow module (42 genes), the green module (34 genes). Additionally, there was a large set of 7,450 unassigned genes classified as the grey module (**Supplementary Figure 1B**). A full list of genes included in each module is provided in **Supplementary Table 3**. Hierarchical clustering of module eigengenes confirmed that all five modules remained distinct with dissimilarity values exceeding the standard WGCNA merging threshold of 0.25 (**Supplementary Figure 1C**). Correlation of module membership with gene significance confirmed enrichment of genes in the yellow and brown modules (**Supplementary Figure 1D**). Module-trait analysis revealed that the brown module exhibited significant correlation with thrombotic PAPS (r=0.209, p=0.046), while the yellow module showed a lower and less statistically significant correlation with PAPS (r=0.187, p=0.076) (**Supplementary Figure 1E**). No other module showed a significant correlation with thrombotic PAPS when compared with HCs.

### Biological functions of the yellow and brown modules

**Figure 1:**
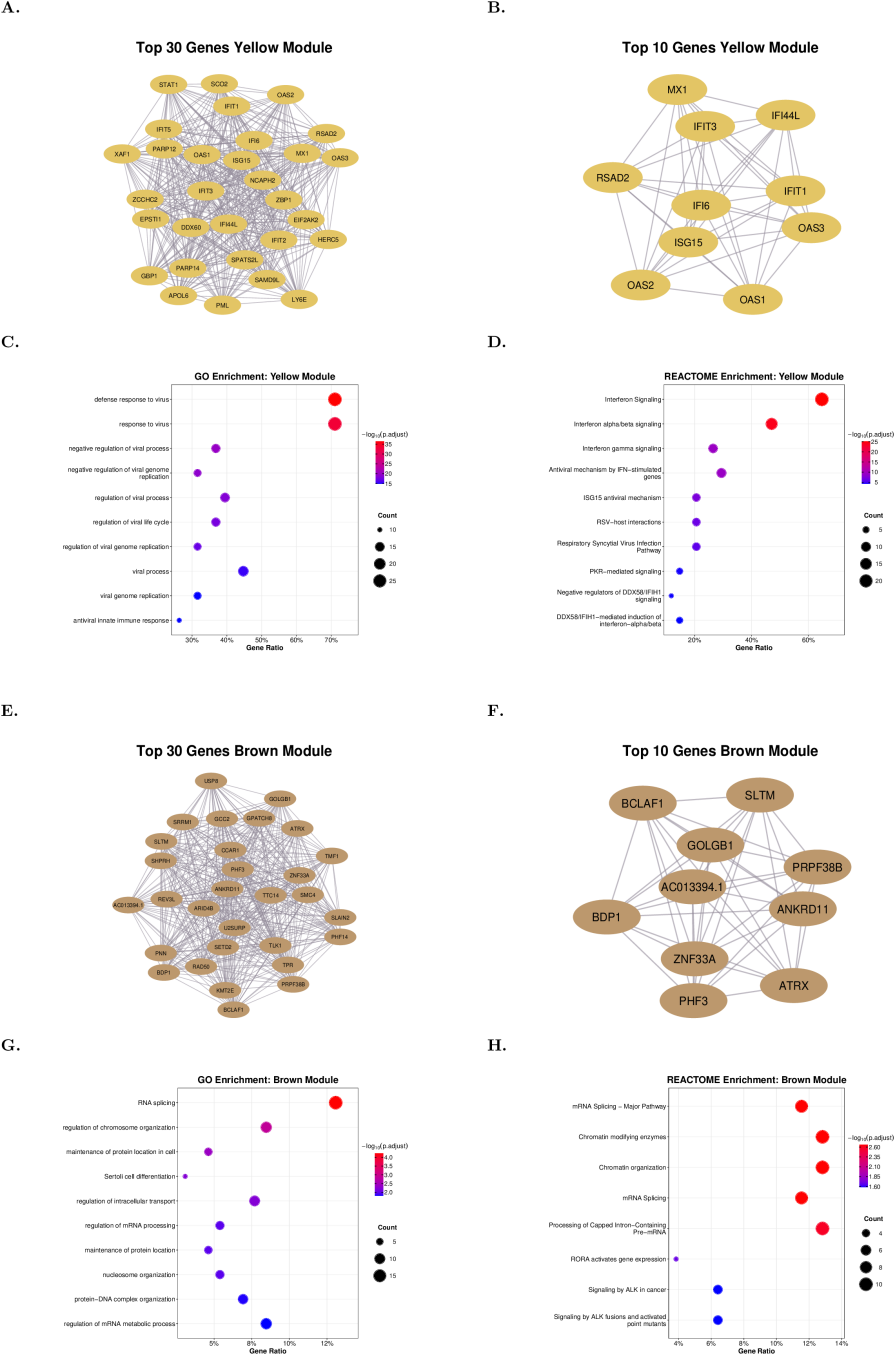
Network topology and functional enrichment of thrombotic PAPS-associated modules. **(A)** Co-expression network of the top 30 hub genes in the yellow module. **(B)** Co-expression network of the top 10 hub genes in the yellow module showing the highest intramodular connectivity. **(C)** Gene Ontology biological process enrichment for the yellow module. **(D)** REACTOME pathway enrichment for the yellow module. **(E)** Co-expression network of the top 30 hub genes in the brown module. **(F)** Co-expression network of the top 10 hub genes in the brown module. **(G)** Gene Ontology biological process enrichment for the brown module. **(H)** REACTOME pathway enrichment for brown module. Dot size indicates gene count; color intensity represents -log10(adjusted p-value). All enrichment analyses used Benjamini-Hochberg correction (p.adjust<0.05).

The investigation of the biological functions of the PAPS-associated yellow module revealed that it contained genes enriched in defense response to viruses. Co-expression networks of the top 30 hub genes are illustrated in **Figure 1A**. The top 10 hub genes of the yellow module were *OAS3, RSAD2, IFI44L, MX1, IFIT1, IFIT3, OAS1, IFI6, OAS2, and ISG15*, with their connectivity depicted in **Figure 1B**. Gene Ontology enrichment analysis for the yellow group has shown a strong association with antiviral defense and immune responses, including regulation and inhibition of viral processes, genome replication, and mainly innate immune mechanisms against viral infection (**Figure 1C**, **Supplementary Table 4**). Reactome enrichment analysis for the yellow group reveals a strong activation of IFN-related and antiviral response pathways, including type I and II IFN signaling, ISG15 (Interferon-Stimulated Gene 15) and PKR (Protein Kinase R)-mediated antiviral mechanisms, and host responses to respiratory viruses such as RSV (**Figure 1D**, **Supplementary Table 5**).

The brown module exhibited a general regulatory profile with genes involved in RNA regulation and transcriptional activation. Co-expression networks of the top 30 hub genes are illustrated in **Figure 1E**. The top 10 hub genes of the brown module were *ATRX, GOLGB1, SLTM, PHF3, ANKRD11, BCLAF1, AC013394.1, PRPF38B, ZNF33A, and BDP1*, shown in **Figure 1F**. Gene Ontology enrichment analysis for the brown group highlights biological processes related to RNA splicing, mRNA regulation, and chromatin organization, alongside functions in maintaining protein localization, intracellular transport, and Sertoli cell differentiation (**Figure 1G**, **Supplementary Table 6**). Reactome enrichment analysis for the brown group indicates enrichment of pathways involved in RNA splicing and chromatin regulation, including major mRNA processing pathways and chromatin-modifying enzymes, as well as transcriptional activation by RORA (Retinoic acid-related orphan receptor alpha) and oncogenic signaling through ALK (Anaplastic Lymphoma Kinase) and its fusion variants (**Figure 1H**, **Supplementary Table 7**).

### Merging modules

**Figure 2:**
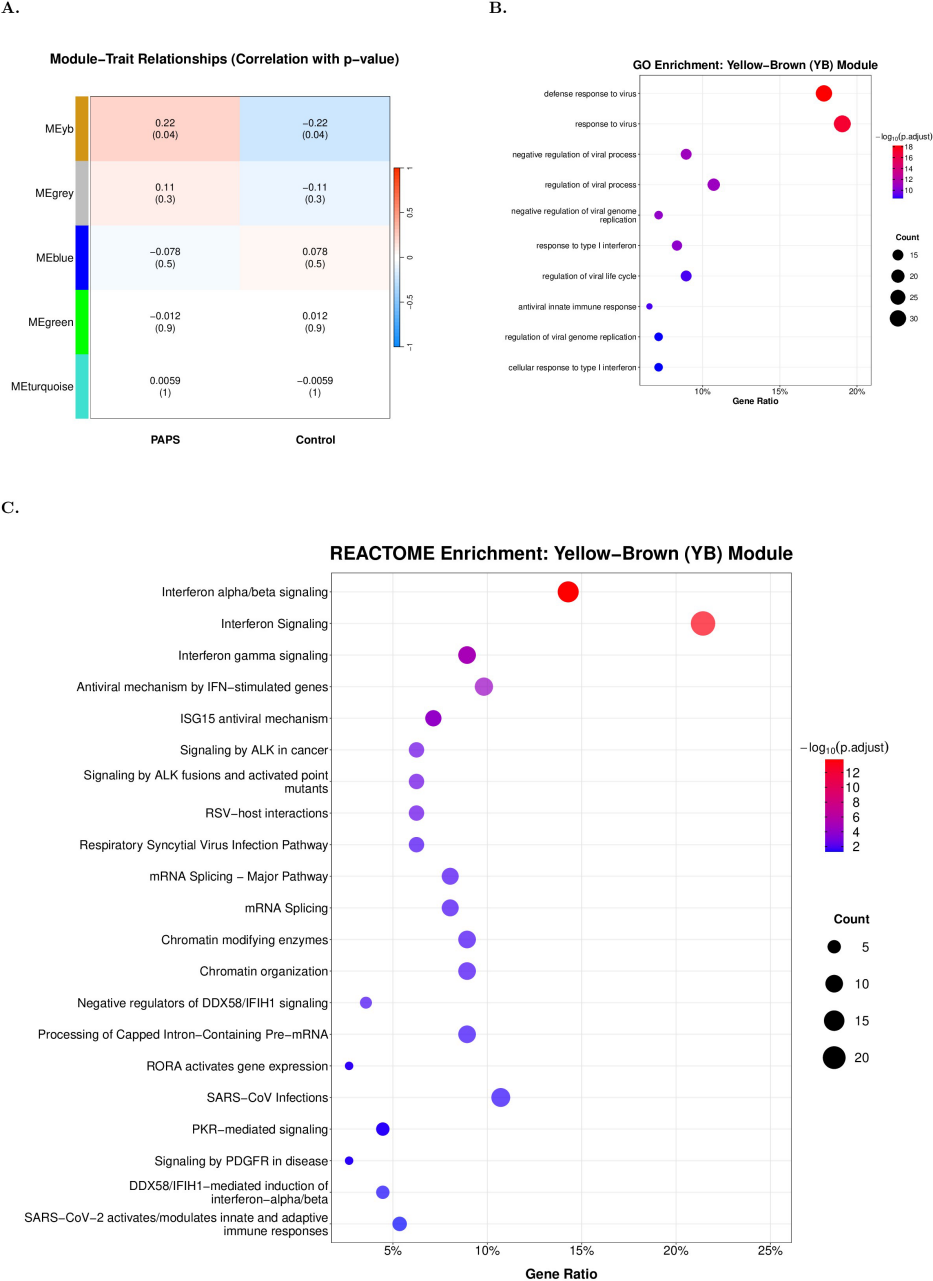
Merged yellow-brown module reveals enhanced association in thrombotic PAPS with antiviral immune signatures. **(A)** Module-trait correlation matrix after merging yellow and brown modules based on eigengene similarity. The merged yellow-brown module (MEyb: 186 genes) shows strengthened association with APS (r=0.221, p=0.035). **(B)** Gene Ontology biological process enrichment for the MEyb module. **(C)** REACTOME pathway enrichment for the MEyb module, showing all significant pathways rather than restricting to top 10 terms. This comprehensive display facilitates identification of the full functional landscape and interconnected biological processes within the module. Six key pathways from this enrichment were selected for color-coded network visualization in **Figure 4**: Interferon alpha/beta signaling, Interferon gamma signaling, ISG15 antiviral mechanism, PKR-mediated signaling, RORA activates gene expression, and Signaling by PDGFR in disease. In B-C, dot size indicates gene count; color intensity represents -log10(adjusted p-value). All enrichment analyses used Benjamini-Hochberg correction (p.adjust<0.05).

Given the exploratory nature of this network analysis and the biological coherence between these modules, we proceeded to investigate both modules. Importantly, merging these functionally related modules yielded a significantly strengthened association (MEyb: r=0.221, p=0.035) than the individual yellow (r=0.187, p=0.076) and brown (r=0.209, p=0.046) modules, supporting the validity of considering both in our analysis (**Figure 2A**). Gene Ontology analysis of MEyb identified response to virus, innate immune response and response to type I IFN as top gene ontology terms (**Figure 2B**). Reactome results for MEyb (**Figure 2C**, **Supplementary Table 8**) revealed pathways related to innate and adaptive immune responses, including IFN signaling, viral infection responses, and their regulation, alongside disease-associated growth factor signaling through PDGFR (platelet-derived growth factor receptor alpha) and PKR. Furthermore, Meyb Reactome pathways related to transcription regulation were found to be enriched. Those pathways included oncogenic ALK-driven signaling, regulation of gene expression through chromatin modification and organization, and control of transcriptional output via RORA activation and RNA processing mechanisms. In summary, the yellow module contained genes enriched in immune response and IFN signaling pathways, while the brown module exhibited enrichment in transcription regulation and RNA processing pathways. The merged yellow-brown module (MEyb), which showed significant association with PAPS (r=0.221, p=0.035), retained enrichment for both IFN-related immune functions and transcriptional regulatory processes, reflecting their coordinated dysregulation in thrombotic PAPS.

**Figure 3:**
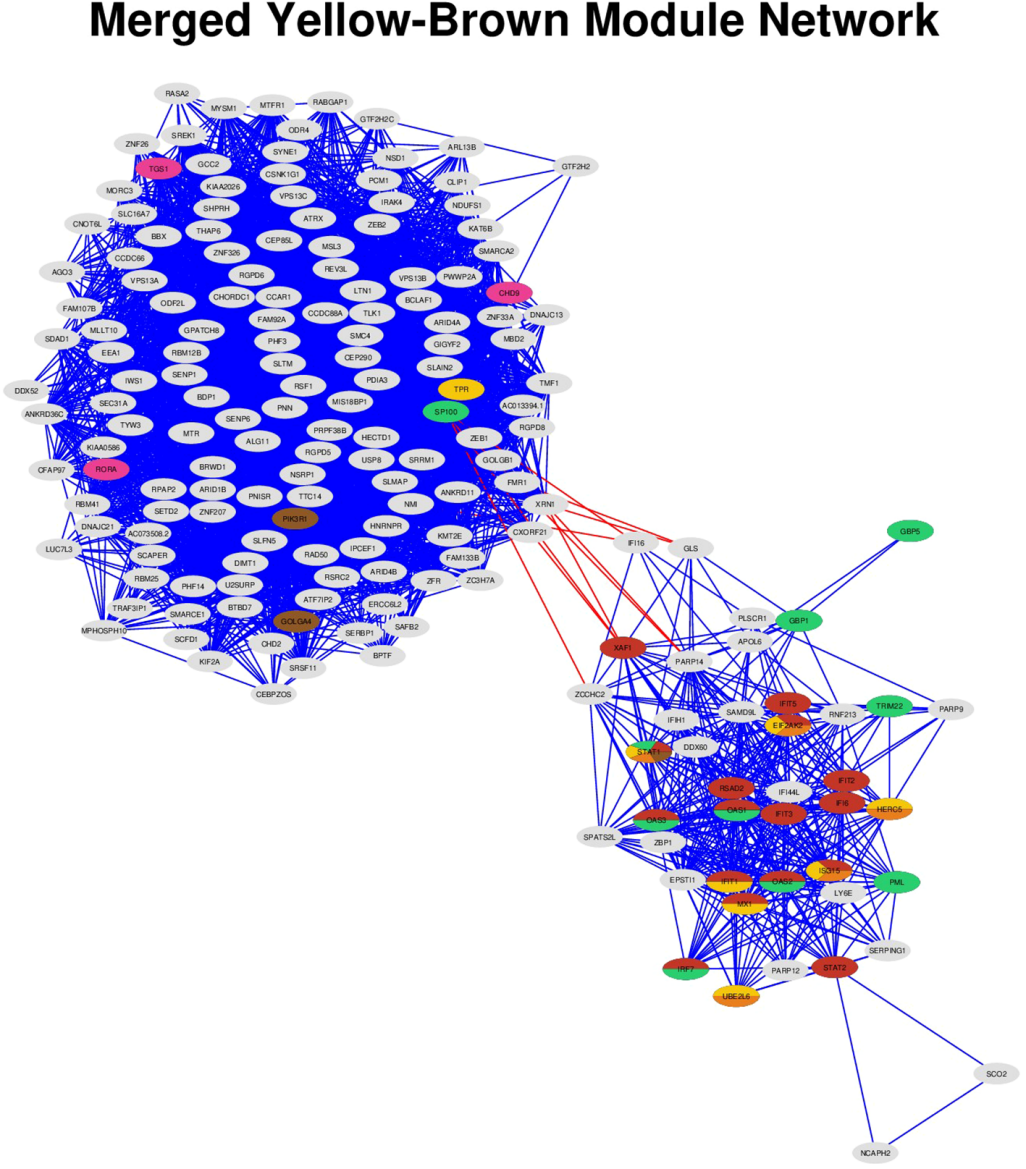
Co-expression network of merged yellow-brown module genes reveals pathway-specific substructure. Co-expression network visualization of the MEyb module (186 genes). Nodes represent individual genes, with pie charts displayed on pathway-annotated genes (29/186) indicating membership across six key enriched REACTOME pathways (see **Figure 2C**). Pathway color coding: Interferon alpha/beta signaling (red), Interferon gamma signaling (green), ISG15 antiviral mechanism (yellow), PKR-mediated signaling (orange), RORA activates gene expression (pink), Signaling by PDGFR in disease (brown). Multi-colored pie slices indicate genes participating in multiple pathways. Edge colors distinguish interaction origins: red edges indicate cross-module connections between the original yellow and brown modules; blue edges show within-module connections. The network displays correlations above the third quartile threshold, emphasizing the strongest co-expression relationships within the merged module.

Then, we focused on six significantly enriched immune-related pathways for detailed network analysis: interferon (IFN) alpha/beta signaling, IFN gamma signaling, ISG15 antiviral mechanism, PKR-mediated signaling, RORA activates gene expression, and signaling by PDGFR. Network visualization of MEyb revealed substantial overlap across the above pathways, with Signal Transducer and Activator of Transcription 1 (*STAT1*) serving as a central hub connecting five of the six pathways (**Figure 3**). Importantly, *STAT1*’s position as a network hub was determined through topology-based analysis of gene co-expression patterns, and its participation in multiple enriched pathways provides convergent functional evidence for its role in bridging innate immune response and transcriptional regulation. Gene-pathway membership matrix and edge list for Cytoscape visualization of the merged yellow-brown module (Q3 threshold applied) are shown in **Supplementary Table 9** (9a. Node annotation file; 9b. Edge list file). This functional integration between the original modules suggests coordinated dysregulation of innate immunity and regulatory processes in PAPS.

### Drug-gene interaction analysis results

The results of the drug-gene interaction analysis, including detailed drug-gene interaction data with specific compound names, interaction types, and classifications for all 186 genes, are provided in **Supplementary Table 10**. Drug-gene interaction of the gene set revealed that the majority had no annotated drug interactions and were classified as low priority. A smaller subset, however, showed evidence of tractability through existing inhibitors or modulators. Among these, *TLK1*(Tousled-Like Kinase1) emerged as the top candidate with four kinase inhibitors and the highest combined scores (drug-gene interaction score 0.67, priority score 0.769), designating it as having high drug-gene interaction annotation and high priority. *PIK3R1* and GLS also ranked highly, each with two inhibitors, moderate drug-gene interaction scores (0.44) but elevated priority scores (0.608), reflecting both their pharmacological relevance and membership in druggable genome categories*. STAT1*, one of the four top-ranked genes, had a drug-gene interaction score of 0.39 and a priority score of 0.573, and displayed many interactions and strong pharmacological support (**Supplementary Table 10**).

**Figure 4:**
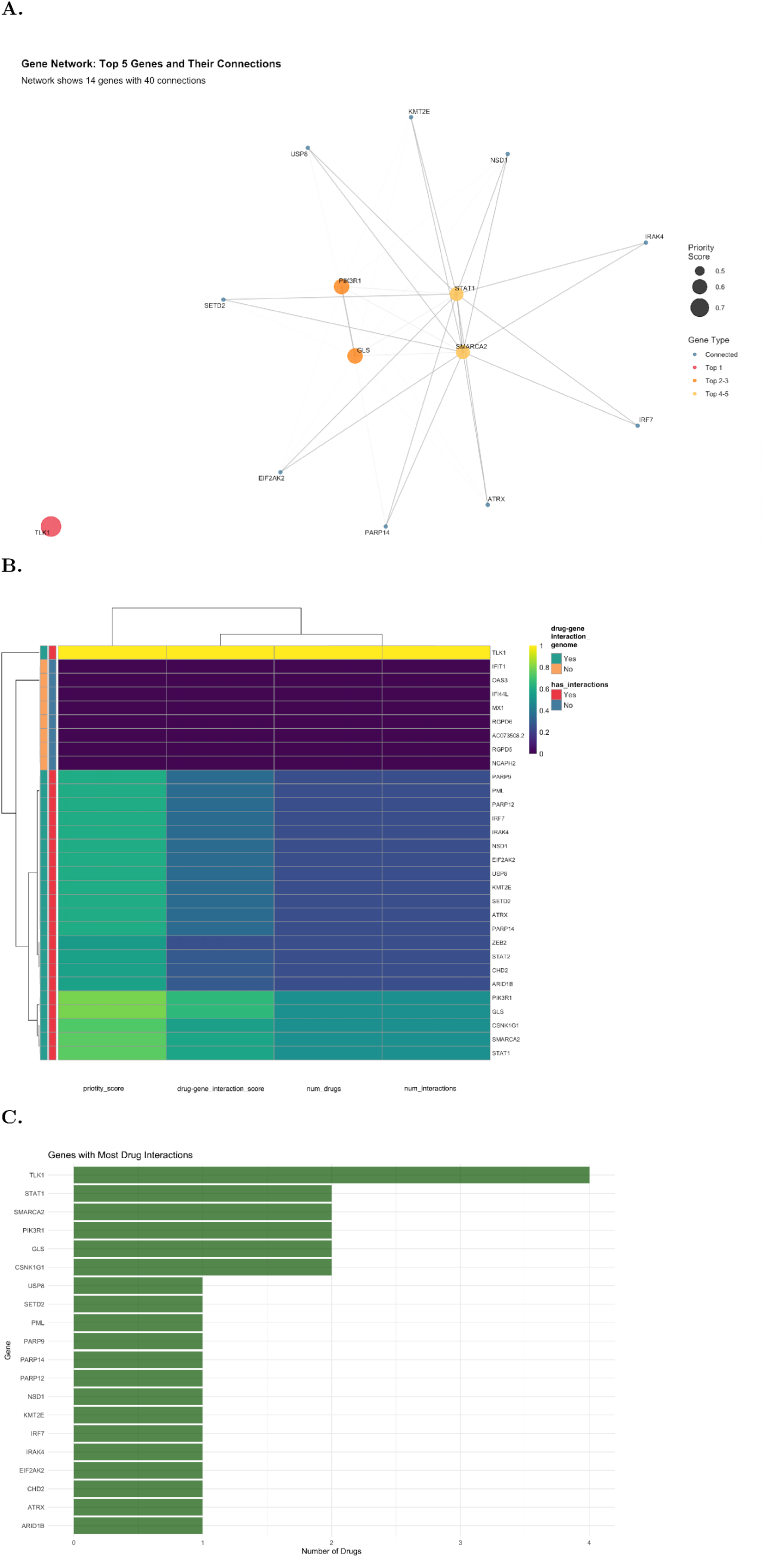
Drug-gene interaction annotation and prioritization of candidate genes. **(A)** Gene interaction network highlighting the top prioritized candidates. Node size reflects the prioritization score, and node color indicates drug-gene interaction classification (low, moderate, or high). **(B)** Heatmap of prioritized genes based on four values: prioritization score, drug-gene interaction score, number of interactions, and number of drugs. Genes are further grouped according to whether they have interactions and if they were characterized as genome annotated. **(C)** Bar plot showing genes with known interactions ranked by the number of targeting agents. The x-axis depicts the number of drugs per gene.

Network visualization of the top-ranked genes (**Figure 4A**) highlighted *TLK1* as a hub non-connected to other compounds, while *PIK3R1* and *GLS* formed secondary nodes with distinct sets of inhibitors. *STAT1* clustered with immune-related signaling mediators, consistent with its known therapeutic targeting in inflammatory pathways. The heatmap of drug-gene interaction and priority scores (**Figure 4B**) reinforced these patterns, separating *TLK1, PIK3R1*, and *GLS* as the highest-priority candidates, while grouping *STAT1, IRAK4, EIF2AK2*, and *PARP* genes into a medium-priority cluster. The remaining genes lacked interactions and occupied a distinct low-priority stratum. The genes with the highest number of target drugs were *TLK1* with 4 candidates, followed by *STAT1, SMARCA2, PIK3R1, GLS, CSNK1G1*, each with two candidates (**Figure 4C**).

Detailed characterization of drug-gene interactions revealed specific therapeutic compounds for the top-ranked candidates (**Supplementary Table 10**). *TLK1* showed interactions with four kinase inhibitors, though these remain investigational compounds. *PIK3R1* demonstrated drug-gene interaction through two FDA-approved PI3K inhibitors: alpelisib and idelalisib, both currently used in oncology. *GLS* is targetable by two glutaminase inhibitors: CB-839 (telaglenastat), used in cancer trials and has shown promise in preclinical autoimmune models, and BPTES, a research compound. STAT1 showed moderate drug-gene interaction with two targeting agents: fludarabine, a direct *STAT1* inhibitor approved for hematological malignancies, and ruxolitinib, a JAK1/JAK2 inhibitor that indirectly modulates *STAT1* signaling. Ruxolitinib has been approved for myeloproliferative neoplasms and is under investigation in several autoimmune disorders.

Among genes with known drug interactions, several showed moderate drug-gene interaction, including *SMARCA2* (targeted by PFI-3 and BAF312), *EIF2AK2/PKR* (2-aminopurine), *IRAK4* (imidazopyridine derivatives), and *IRF7* (BX795). Multiple *PARP* family members (*PARP9, PARP12, PARP14*) were identified as druggable through PARP inhibitors such as olaparib, though their therapeutic relevance in PAPS remains uncertain. The majority of genes (157 of 186) in the MEyb module lacked annotated drug interactions.

## Discussion

APS is a complex and life-threatening autoimmune disease with an incompletely defined pathogenesis. This is the first study to our knowledge that performed network gene co-expression analysis and drug-gene interaction analysis on whole-blood RNA-seq data from patients with thrombotic PAPS and age- and sex-matched HCs.

Applying WGCNA, we identified five gene co-expression networks, two of which reached a significant correlation with thrombotic PAPS compared to HCs. The genes in the yellow module were involved in immune response functions and IFN signaling. The brown module contained genes enriched in mRNA splicing machinery, chromatin remodeling, and transcriptional regulation. The merged yellow-brown MEyb was significantly associated with PAPS and retained enrichment for both IFN-related and transcriptional regulatory processes. In accordance with our findings, one of the earliest studies examining gene expression profiles in peripheral blood mononuclear cells (PBMCs) from PAPS patients found that the top gene ontology category included transcription factors, coactivators and repressors, splicing factors and chromatin structure regulators. Additionally, five out of nine overrepresented categories were related to innate immune responses. Type I IFN-induced genes (*LGALS3BP, WARS, OAS2, G1P2, GTPBP2, TNFSF13, PLSCR1, H1F0, and STAT1*) and type II IFN-regulated genes were included in these categories (27). Further research demonstrated type I IFN (IFN-I) pathway activation in isolated monocytes, PBMCs or whole blood of patients with PAPS compared to HCs (22, 28, 29). Notably, an overall activation of the IFN-I pathway has been described across the entire APS spectrum (aPL carriers, PAPS, secondary APS), with differences among genes based on specific disease subsets (29). IFN-I signature in the above studies correlated with specific clinical features such as earlier disease onset and preeclampsia, as well as laboratory markers like anti-β2GPI antibodies, and may indicate a worse prognosis (30, 31). Significantly increased expression of type I and type II IFN-regulated genes has also been identified in transcriptomic studies in the monocytes, neutrophils, whole blood and kidney biopsies of PAPS patients (3, 11, 19, 32) and a recent proteomic study including patients with thrombotic PAPS (33). While IFN signaling genes are primarily located in the yellow module in our study, their co-expression and shared regulation with transcriptional machinery genes in the brown module through the STAT1 hub reflect the mechanistic integration of immune signaling with transcriptional output in PAPS pathogenesis.

In our study, while IFN signaling genes are primarily located in the yellow module, they are co-expressed and share regulation with transcriptional machinery genes in the brown module through the STAT1 hub, which was identified as chief regulator. S*TAT1* plays a central role in mediating the effects of IFNs, including type I IFNs (IFN-α and IFN-β), type II IFN (IFN-γ), and type III IFNs, by transmitting signals from cytokine receptors to the cell’s nucleus to trigger gene expression. After IFNs bind to their receptors, *STAT1* is phosphorylated by Janus kinases (JAKs) and Tyrosine Kinase 2 (TYK2), leading to its dimerization and translocation into the nucleus. In the nucleus, *STAT1* activates the transcription of IFN-stimulated genes, which coordinate diverse biological effects including antiviral defense, antigen presentation, immune cell activation, and inhibition of cell proliferation (34). Bernales et al. described significantly increased STAT1 gene expression profiles in PBMCs from PAPS patients (27). In a multi-center genome-wide association study in PAPS, the most significant genetic association corresponded to avariant (rs11889341) located in STAT1-STAT4 (35). While the central role of STAT1 in IFN signaling is well-established in autoimmune diseases, our unbiased network analysis provides data-driven validation of its specific importance in thrombotic PAPS pathogenesis and, critically, integrates this biological knowledge with drug-gene interaction assessment to identify actionable therapeutic strategies. The convergence of network topology, functional enrichment and pharmacological tractability on STAT1 as a central, druggable hub supports its potential as a therapeutic target in thrombotic PAPS.

Assessment of drug-gene interactions for the candidate gene set showed that most genes lacked annotated drug interactions and were classified as of low priority. A subset of genes, however, demonstrated tractability through existing inhibitors or modulators. *TLK1* emerged as the top candidate, with four kinase inhibitors and the highest combined drug-gene interaction and priority scores, indicating therapeutic potential. *PIK3R1* and *GLS* also ranked highly, each with two inhibitors and moderate drug-gene interaction, and *STAT1*, which was also ranked highly, showed multiple interactions and strong pharmacological support, placing it in a medium-priority category. Overall, based on existing drug-gene interaction data, these results highlight *TLK1, PIK3R1, GLS* and *STAT1* as genes with established pharmacological targeting options that warrant further investigation for therapeutic intervention.

*TLKI-1* is involved in chromatin organization, DNA repair, transcription, and chromosome segregation. Inhibiting *TLK1* can disrupt this process, making cancer cells more vulnerable to DNA damage, however, its role in autoimmune disorders has not been elucidated. The *PIK3R1* gene provides instructions for making a protein that is a regulatory subunit of a cell-signaling molecule called phosphoinositide 3-kinase (PI3K). *PI3K* plays a crucial role in immune responses by regulating immune cell growth, survival, differentiation, and function. The role of PI3K in T cell-dependent humoral immune responses and B cell homeostasis, and its aberrant activity in a range of autoimmune diseases such as SLE, rheumatoid arthritis, and multiple sclerosis, supported a rationale for the development of PI3K inhibitors in autoimmune disorders (9, 36, 37). In preclinical models, inhibition of PI3Kγ has shown promise for rheumatoid arthritis and SLE treatment. The dual inhibition of PI3K delta and gamma reduced chronic B cell activation and autoantibody production in mouse models of lupus (38, 39). Clinical trials testing new-generation PI3Kγ inhibitors in human autoimmune and inflammatory diseases have not yet been initiated. The *GLS* gene, with its two main isoforms, *GLS1* and *GLS2*, is crucial in immune cell function and differentiation through its role in glutamine metabolism. GLS expression affects the expression of other immunomodulatory genes and is linked to the activity of both innate and adaptive immunity (40). *GLS* plays a role in the metabolism of immune cells, particularly T-lymphocytes, and is critical for their activation and proliferation. *GLS1* is essential for the differentiation of Th17 cells, a subset of T helper cells that plays a significant role in driving autoimmune diseases, like SLE (41).

A critical consideration in translating our findings to therapeutic strategies is integrating network centrality with pharmacological target availability Drug-gene interaction annotation in our study revealed several potential drugs interfering with dysregulated genes within the IFN-signaling modules, particularly *STAT1*. Therapies targeting IFN have been tested in several systemic autoimmune diseases, aiming to neutralize IFN-α, block its downstream effects by targeting the type I IFN receptor, or inhibit its production (42). Interestingly, in one of the earliest studies reporting on IFN-I activity in PAPS, endothelial progenitor dysfunction was successfully reversed by a type I IFN receptor-neutralising antibody (43). Targeting the IFN gene signaling pathway by downstream signaling pathways (JAK-STAT) is under investigation in patients with systemic autoimmune disease, including SLE (44, 45). In our study, although STAT1 was classified as moderate priority (score: 0.573), it represents a compelling therapeutic target due to its position as a central hub connecting five of six enriched immune pathways and the availability of clinically relevant modulators. STAT1 activity can be modulated through direct inhibitors (fludarabine) or indirectly by JAK inhibitors that prevent STAT1 phosphorylation (ruxolitinib, tofacitinib, baricitinib) (46). Baricitinib has shown efficacy in SLE clinical trials and is approved for rheumatoid arthritis, making it a particularly relevant candidate for repurposing in PAPS given the shared IFN signature. PIK3R1 (targeted by alpelisib, idelalisib) and GLS (CB-839) represent additional opportunities, with CB-839 showing efficacy in suppressing Th17 differentiation in preclinical lupus models (47). Most IFN-regulated genes in the yellow module (OAS3, RSAD2, IFI44L, MX1, IFIT family) showed limited available drug-gene interactions in current databases, lacking enzymatic or structural features amenable to small molecule inhibition. This reinforces the strategic importance of targeting upstream regulators like STAT1 and JAKs, which can modulate the entire IFN signature through pathway-level intervention. JAK/STAT inhibitors offer repurposing potential, leveraging existing safety data from other autoimmune diseases.

One of the strengths of our study is the large number of well-characterised PAPS patients, considering the rarity of the syndrome, and the inclusion of a homogeneous group of PAPS patients with thrombotic events. APS is a highly heterogeneous disorder that may occur in its primary form (PAPS) or in association with another autoimmune disease, mainly SLE (SLE/APS). It can also present with a wide range of clinical manifestations, including thrombotic manifestations, obstetric complications, as well as microvascular, cardiac and hematological manifestations. A major strength of the study is the inclusion of only PAPS patients, excluding those with coexistent SLE or other autoimmune disorders. This helps avoid any confounding factors from the IFN and STAT1 signature present in these disorders. Notably, none of the patients met the SLE classification criteria during their disease course (mean disease duration: 7.62 +/-7.58) and their follow-up after their enrolment in the study. Thirty-five percent of patients with PAPS tested positive for antinuclear antibodies (ANA) (all but three had lower than 1/640 titres), however, none of them had low complement (C3 or C4) levels, positive anti-dsDNA or anti-Sm antibodies. In a recent study using data from the AntiPhospholipid Syndrome Alliance for Clinical Trials and InternatiOnal Networking (APS-ACTION) registry, ANA were detected in 44% of patients with PAPS (48). Our study also has some limitations, such as the inclusion of only White individuals, which reduces the generalizability of our findings to more diverse ethnic groups. Our drug-gene interaction analysis provides a database-based annotation of known drug–gene interactions and target availability. Experimental validation including perturbational studies and assessment of whether candidate drugs can reverse the disease-related gene expression signature will help to confirm therapeutic potentials. Furthermore, the computational drug-gene interaction predictions do not account for safety profiles in thrombotic diseases, potential prothrombotic effects of immunosuppression or complex drug-disease interactions specific to PAPS. Future validation will require preclinical studies in APS mouse models, ex vivo studies on patient-derived cells and clinical pilot studies of approved drugs in refractory PAPS patients. In conclusion, gene co-expression network and drug-gene interaction analysis in our study have helped identify novel potential therapeutic targets for patients with thrombotic PAPS. Future work integrating transcriptomic findings with functional validation studies, including ex vivo drug treatment experiments in patient-derived cells, would enable assessment of how candidate drugs reverse the molecular signatures observed in PAPS.

## Data Availability

The complete RNA-sequencing dataset, including raw counts and normalized expression matrices, has been deposited in the Gene Expression Omnibus (GEO) database under accession number GSE205465. The code used for data processing and analysis is available at GitHub: https://github.com/michalisbalts/APS_WGCNA.

## Supplementary material

Supplementary Figure 1 Weighted gene co-expression network analysis identifies PAPS-associated gene modules. (A) Hierarchical clustering dendrogram of 8,190 genes based on topological overlap dissimilarity. Modules were identified using dynamic tree cutting followed by merging of similar modules (dissimilarity threshold <0.25), resulting in five final modules across 91 samples (62 thrombotic PAPS patients, 29 controls). (B) Distribution of genes across modules: grey (7,450 unassigned), turquoise (349), blue (171), brown (144), yellow (42), and green (34). (C) Clustering of module eigengenes showing eigengene dissimilarity among modules; the red dashed line indicates the standard WGCNA merging threshold (dissimilarity = 0.25). (D) Module membership versus gene significance for thrombotic PAPS association, showing correlations between intramodular connectivity and trait association strength. (E) Module-trait correlation matrix. Brown and yellow modules show positive associations with thrombotic PAPS (MEbrown: r=0.209, p=0.046; MEyellow: r=0.187, p=0.076).

Supplementary Table 1 Patient characteristics.

Supplementary Table 2 Filtered and VST-normalized expression matrix – Expression values for 8,190 quality-filtered genes across 91 samples.

Supplementary Table 3 Module gene lists – Complete gene assignments for all WGCNA modules (ordered by descending module size). 3a. Turquoise module (349 genes); 3b. Blue module (171 genes); 3c. Brown module (144 genes); 3d. Yellow module (42 genes); 3e. Green module (34 genes)

Supplementary Table 4 Yellow module GO enrichment – Gene Ontology enrichment results for the yellow module.

Supplementary Table 5 Yellow module REACTOME pathways – REACTOME pathway enrichment for the yellow module.

Supplementary Table 6 Brown module GO enrichment – Gene Ontology enrichment results for the brown module.

Supplementary Table 7 Brown module REACTOME pathways – REACTOME pathway enrichment for the brown module.

Supplementary Table 8 Merged yellow-brown module REACTOME pathways – REACTOME pathway enrichment results for the merged yellow-brown module.

Supplementary Table 9 Network visualization data – Gene-pathway membership matrix and edge list for Cytoscape visualization of the merged yellow-brown module (Q3 threshold applied).9a. Node annotation file; 9b. Edge list file.

Supplementary Table 10 DGIdb drug-gene interaction annotation results – Drug-gene interaction assessment for merged yellow-brown module genes.
