# Supplementary figures and images for: RNA sequencing-derived gene co-expression and drug-gene interaction analysis reveal STAT1 as a potential therapeutic target in thrombotic antiphospholipid syndrome
Source: Front Immunol. 2026 Mar 5;17:1741872. doi: 10.3389/fimmu.2026.1741872 (PMC12999812; doi:10.3389/fimmu.2026.1741872)

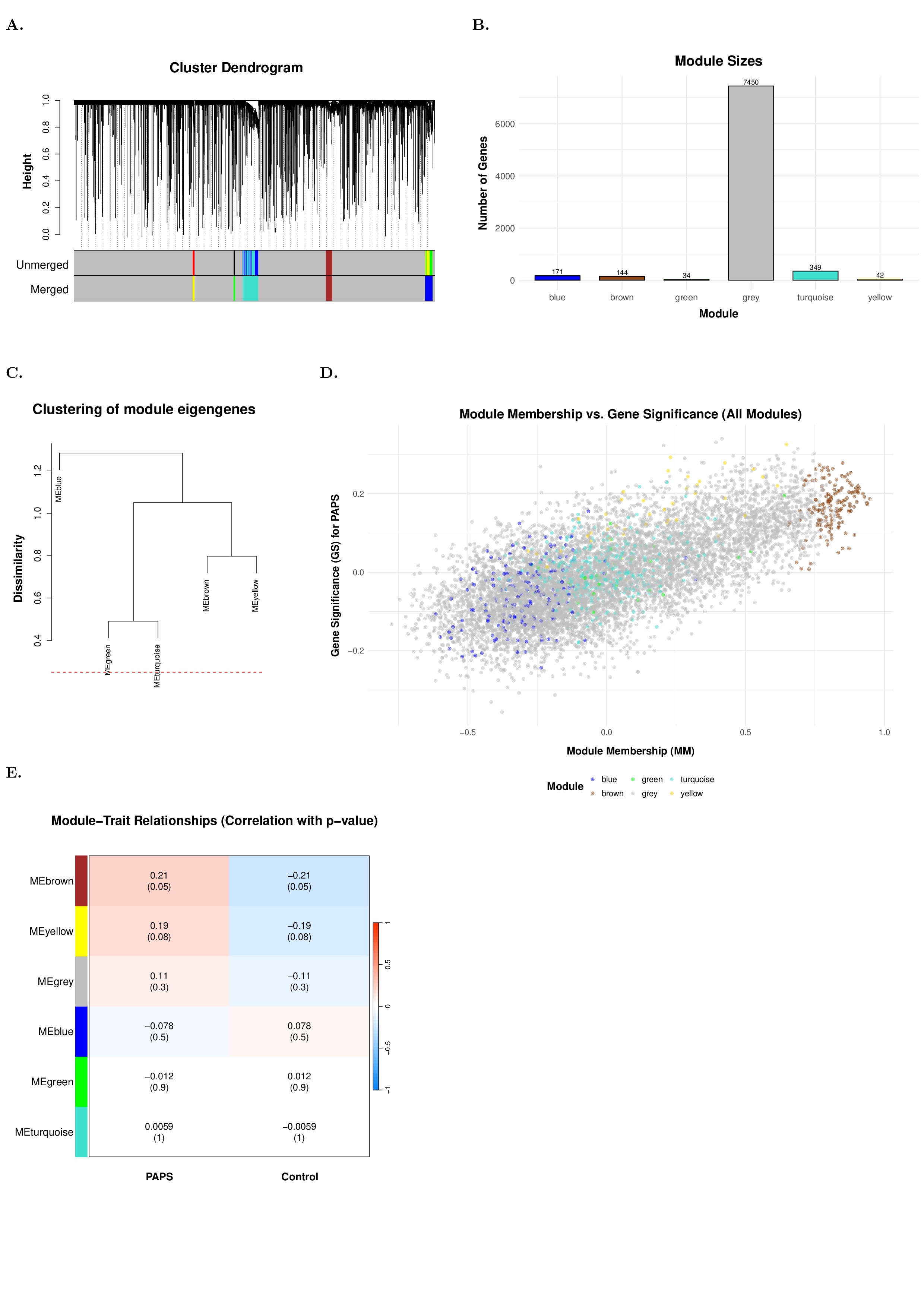

Supplement: Supplementary Figure 1 — Weighted gene co-expression network analysis identifies PAPS-associated gene modules. (A) Hierarchical clustering dendrogram of 8,190 genes based on topological overlap dissimilarity. Modules were identified using dynamic tree cutting followed by merging of similar modules (dissimilarity threshold <0.25), resulting in five final modules across 91 samples (62 thrombotic PAPS patients, 29 controls). (B) Distribution of genes across modules: grey (7,450 unassigned), turquoise (349), blue (171), brown (144), yellow (42), and green (34). (C) Clustering of module eigengenes showing eigengene dissimilarity among modules; the red dashed line indicates the standard WGCNA merging threshold (dissimilarity = 0.25). (D) Module membership versus gene significance for thrombotic PAPS association, showing correlations between intramodular connectivity and trait association strength. (E) Module-trait correlation matrix. Brown and yellow modules show positive associations with thrombotic PAPS (MEbrown: r=0.209, p=0.046; MEyellow: r=0.187, p=0.076). [file Image1.jpeg]
