# Supplementary material for: RNA sequencing-derived gene co-expression and drug-gene interaction analysis reveal STAT1 as a potential therapeutic target in thrombotic antiphospholipid syndrome
Source: Front Immunol. 2026 Mar 5;17:1741872. doi: 10.3389/fimmu.2026.1741872 (PMC12999812; doi:10.3389/fimmu.2026.1741872)
Supplement: Supplementary Table 1 — Patient characteristics. [file Table1.docx]

**Supplementary Table 1**. Patient characteristics (N=62)

| **Patient characteristics** | **Thrombotic primary APS**  **N=62** |
| --- | --- |
| Female, n (%) | 40 (64.5%) |
| Mean age, years; mean (S.D.) range | 42.5 (12.36) (18, 69) |
| Disease duration; mean (S.D.) range | 7.62 (7.58) (0, 28) |
| Venous thrombosis only, n (%) | 29/62 (46.8%) |
| Arterial thrombosis only, n (%) | 21/62 (33.8%) |
| Venous and arterial thrombosis, n (%) | 11/62 (17.7%) |
| Recurrent thrombosis, n (%) | 25/62 (40.3%) |
| Anticardiolipin antibodies (IgG or IgM) | 50/62 (80.6%) |
| Single (moderate/high) anticardiolipin antibody positivity | 3/62 (3.22%)  (1 patient with IgG and IgM anticardiolipin antibodies, 2 patients with IgG and IgM anticardiolipin antibodies) |
| Anticardiolipin antibodies, IgG | 31/62 (50%) |
| Anticardiolipin antibodies, IgM | 40/62 (64.5%) |
| Anti-beta2glycoprotein I (IgG or IgM) | 39/62 (62.9%) |
| Single (moderate/high) anti-beta2glycoprotein I antibodies | 3/62 (3.22%)  (1 patient with IgG anti-beta2glycoprotein antibodies alone, 2 patients with IgG and IgM anti-beta2glycoprotein antibodies) |
| Anti-beta2glycoprotein I, IgG | 33/62 (53.2%) |
| Anti-beta2glycoprotein I, IgM | 26/62 (41.9%) |
| Lupus anticoagulant | 49/60 (81.6%) |
| Single Lupus anticoagulant positivity | 10/60 (16.6%) |
| Double antiphospholipid antibody positivity | 47/60 (78.3%) |
| Double antiphospholipid antibody patients excluding those who are also triple positive | 16/60 (26.6%) |
| Triple antiphospholipid antibody positivity | 31/60 (51.6%) |
| C3 levels (S.D.) (range) | 102.4 (25) (62, 185) |
| C4 levels (S.D.) (range) | 18.7 (8) (2, 44) |
| Low C3 or C4 levels, n (%) | 4/62 (6.4%) |
| Antinuclear antibodies* | 22/62 (35.4%) |
| Anti-dsDNA antibodies | 0/62 |
| Anti-Sm antibodies | 0/60 |
| Direct Coombs test positivity** | 1/59 |
| Hypertension | 15/62 (24.2%) |
| Dyslipidemia | 10/62 (16.1%) |
| Smoking ever | 35/62 (56.4%) |
| Smoking current | 20/62 (32.2%) |
| Smoking (pack years) | 10.76 (14.77) (0, 50) |
| Family History of coronary disease | 6/61 (9.83%) |
| Hydroxychloroquine treatment | 26/62 (42%) |
| Aspirin treatment | 29/62 (46.7%) |
| Anticoagulants | 62/62 (100%) |
| Anti-hypertensive drugs | 14/62 (22.5%) |
| Statin treatment | 10/62 (16.1%) |

*All but three patients with positive antinuclear antibodies had lower than 1/640 titres. None of those with positive antinuclear antibodies had low C3 or C4 complement levels.

** The patient with direct Coombs test positivity was negative for antinuclear antibodies.
